# Implementation of Cyber-Physical Production Systems for Quality Prediction and Operation Control in Metal Casting

**DOI:** 10.3390/s18051428

**Published:** 2018-05-04

**Authors:** JuneHyuck Lee, Sang Do Noh, Hyun-Jung Kim, Yong-Shin Kang

**Affiliations:** Department of Systems Management Engineering, Sungkyunkwan University, 2066 Seobu-ro, Jangan-gu, Suwon, Gyeonggi-do 16419, Korea; kkomang92@skku.edu (J.L.); sdnoh@skku.edu (S.D.N.); kim.hj@skku.edu (H.-J.K.)

**Keywords:** CPPS, cyber-physical production system, big data, metal casting

## Abstract

The prediction of internal defects of metal casting immediately after the casting process saves unnecessary time and money by reducing the amount of inputs into the next stage, such as the machining process, and enables flexible scheduling. Cyber-physical production systems (CPPS) perfectly fulfill the aforementioned requirements. This study deals with the implementation of CPPS in a real factory to predict the quality of metal casting and operation control. First, a CPPS architecture framework for quality prediction and operation control in metal-casting production was designed. The framework describes collaboration among internet of things (IoT), artificial intelligence, simulations, manufacturing execution systems, and advanced planning and scheduling systems. Subsequently, the implementation of the CPPS in actual plants is described. Temperature is a major factor that affects casting quality, and thus, temperature sensors and IoT communication devices were attached to casting machines. The well-known NoSQL database, HBase and the high-speed processing/analysis tool, Spark, are used for IoT repository and data pre-processing, respectively. Many machine learning algorithms such as decision tree, random forest, artificial neural network, and support vector machine were used for quality prediction and compared with R software. Finally, the operation of the entire system is demonstrated through a CPPS dashboard. In an era in which most CPPS-related studies are conducted on high-level abstract models, this study describes more specific architectural frameworks, use cases, usable software, and analytical methodologies. In addition, this study verifies the usefulness of CPPS by estimating quantitative effects. This is expected to contribute to the proliferation of CPPS in the industry.

## 1. Introduction

Today, due to short product lead times, small-quantity batch production, diversification of consumer needs, and irregular demand fluctuations, manufacturing companies are trying to achieve innovation, such as flexible and predictive production, in contrast to the mass production that is a typical manufacturing method [1,2]. In terms of technologies that support manufacturing innovation, information and communication technologies (ICT) including enterprise resource planning, manufacturing execution systems (MES), and programmable logic controller automated factories significantly improve productivity. However, they are unable to satisfy current production needs such as reducing manufacturing lead times and producing small and customized products [3]. Therefore, beyond the third industrial revolution based on electronics and ICT, the fourth industrial revolution is emerging and is centered on Internet of things (IoT), big data, and artificial intelligence (AI).

The newly emerging fourth industrial revolution commenced in Germany and is termed as Industrie 4.0. Along with Germany, several advanced countries are actively pursuing studies in the area of manufacturing [4]. The primary goal of Industrie 4.0 is to build advanced smart factories by combining various ICT tools with typical manufacturing methods [4,5]. The basic characteristics of smart factories include smart networking based on IoT technologies (such as wireless or smart sensor/smart actuators), uncertainty of various factory situations based on big data, and the flexibility of consumer needs [6]. Smart factories allow the collection of massive amounts of in-plant data through real-time synchronization of the factory components and information systems, and they also improve quality and productivity through smart and flexible responses to abnormal situations that occur in a plant [5,6,7].

A variety of technologies are required to build smart factories. Among these technologies, the cyber-physical system (CPS) is an extremely promising technology of Industrie 4.0 and an essential component of a smart factory. Specifically, CPS is composed of collaborating computational entities that connect the cyber world with the surrounding physical environments or processes through data access in an internet environment [5]. A CPS includes embedded systems (such as equipment, buildings, means of transportation, and medical devices), internet services, logistic, coordination, and management processes [8]. The concept of a cyber-physical production system (CPPS) is a manufacturing-centered version of a CPS that fuses computer science (CS), ICT, and manufacturing-science technology [9]. The introduction of a CPPS allows the construction of smart factories that aid in various decision-making processes by predicting the future based on past and present situations [7,10].

Along with the CPPS, a few core technologies required to build a smart factory are IoT, big data, and AI. The IoT refers to a wireless communication capability integrated with sensors and computing that allows the collection of data related to uniquely identifiable objects through the internet [11,12]. Previous studies indicated that the common components of CPS or CPPS include physical-mechanical systems such as factories, machines and products, sensors and operating systems, electronic hardware, software, and digital twins connecting the real and virtual worlds [6,9,13]. Big data refers to a new computing paradigm that allows the collection, processing, and analysis of fast, diverse and massive amounts of data. Massive amounts of structured and unstructured data related to the physical systems of factories are processed/stored through the IoT, and faster and more accurate quality and productivity prediction models are created by using parallel processing support for the existing AI algorithms.

Conversely, although CPS, CPPS, IoT, big data, and AI are the core technologies in smart factories, most studies have examined high-level reference models [11,12,13,14], including the architecture of the smart factories and CPS. There is a paucity of studies that examine concrete fusion and, particularly, the implementation of the aforementioned technologies. Meanwhile, extant studies related to CPS/CPPS implementation have predicted the life or health of machines and tools that are traditional CPS targets and demonstrated the usefulness of CPS/CPPS through a standalone system or laboratory-level test bed [15,16,17,18,19].

Thus, the present study introduces the implementation of a CPPS for real-time quality prediction and operation control at an actual factory. The target factory produces pistons that are used as components in vehicle engines, and the main process involves metal mold casting. In the metal mold-casting process, the internal quality of products due to the casting process is known in the subsequent process, and thus it is difficult to achieve quality verification and management at the moment of casting. In the factory, the internal quality problem of products occurring in the casting process is only detected in the next process step, machining process, and those defects are scrapped after finishing the step. If it is possible to predict the internal quality problem in the casting process with various technologies implemented in the CPPS, it can significantly improve both quality of products and productivity of the factory. Although the study does not address all manufacturing processes and operational issues, it provides a more realistic understanding of how the CPPS that is composed of multiple components is organically connected and its effect in terms of manufacturing operations.

The remainder of the study is organized as follows. Section 2 describes studies conducted on CPS and big data in manufacturing industry. Section 3 defines the CPPS architecture framework for quality prediction and operation control. Section 4 details the application and implementation of the CPPS architecture framework proposed in Section 3 as applied in actual factories. Finally, Section 5 summarizes and describes the significance of the present study.

## 2. Related Research

### 2.1. Cyber-Physical Systems in Manufacturing

Most studies conducted on a CPS or CPPS in manufacturing industry have focused on the architecture and reference models for application to a manufacturing system. This subsection presents studies conducted on reference models related to a CPS, studies conducted on a digital twin that is a core component of a CPS, and actual industry applications of CPS or CPPS.

Studies on CPS reference models described the role of a CPS in Industrie 4.0 and various architectures showing the connection between CPS components. Jazdi [5] defined the main characteristics of a CPS in relation to Industrie 4.0 and presented the connection between a CPS, smart sensors, actuators, and the Cloud in the form of the system architecture. Lee et al. [13] proposed an architecture model that was composed of five levels to build a CPS in a manufacturing system based on Industrie 4.0 and presented phased guidelines for the development and construction of a CPS in smart factories. Monostori et al. [14] defined the maturity of the components required to construct a CPS in a stage-by-stage manner and presented a phased case based on the application of a CPS in manufacturing industry. Bagheri et al. [12] proposed a unified framework for integrating CPS in manufacturing. They also described adaptive clustering methods for interconnected systems and demonstrated self-aware machines integrated by using CPS.

Subsequently, studies related to a digital twin that is a component of a CPS presented either data schemes for the application of a digital twin to actual factories and processes or the necessity and effects of a digital twin. Lee et al. [7] defined a data schema of the components of a digital twin to construct a big data-based prediction of a manufacturing industry by using a CPS. Rosen et al. [20] defined the role and significance of a digital twin in future manufacturing industry by comparing a digital-twin based design with a design based on a simple simulation. Gabor et al. [21] presented an architectural framework of a digital twin based on a simulation linked to the physical world inside a CPS configuration environment and defined the digital twin instance models and classes required by the architectural framework. Uhlemann et al. [22] proposed a method to minimize the digital twin generation time delay through a multimodal data acquisition approach. They also presented concepts for the composition of the database and guidelines for implementing digital twins in small business production systems.

The last involves the study of the practical implementation of CPS or CPPS. Specifically, we investigated cases related to the environment by using IoT/sensor or predictive manufacturing systems. Morgan and O’Donnell [15] applied CPS to the computerized numerical control (CNC) turning process to form a system to monitor the measurement of process conditions by using sensors in real time. They applied cyber-physical process monitoring systems to the CNC turning process to achieve a series of internal and external individual process analyses by using vibration and electric motor current data. Lee et al. [16] presented CPS frameworks for predictive manufacturing systems and validated the same by applying them to ball-screw prognostics. They verified the predictability of a cyber-physical system to ball-screw prognostics to reduce unplanned downtime through predictive analytics of the components of the process. Guo et al. [17] studied the implementation and verification of a basic diagnostic system for failure detection with CPS. They proposed a cyber-physical failure-detection model by using a data-fusion algorithm and tested the performance metrics for fusion effects on the bandwidth of a mailbox. Alippi et al. [18] investigated the implementation of technologies to detect changes in sensor acquisition by CPS units from the perspective of self-adaptive cyber-physical systems (CPSs) at the sensor level. In order to apply a suitable configuration for embedded systems, they used an augmented reality (AR) predictive model, the ICT-based change-detection test working in the residual space, and a change-point method with the aim of reducing the occurrence of false positive detections and providing an estimate of the time instant when the change occurred, and they validated this in a laboratory environment. He et al. [19] designed cyber-physical test beds to access the performance of mechanical design and system implementation. They used the ultra-wide band channel emulator and body sensors to validate the test beds and argued that it aids in virtualizing the environment of wireless access and localizing the body sensor network. Chen et al. [23] proposed a method for the creation of a CPS model of a CNC machine tool based on electronic data analysis. They collected and analyzed work task data, manufacturing-resources data, and operation status data in order to accurately represent the relationship between input and output variables in the CPS model of the CNC machine tool while it was working. They also argued that task status data can be used to realize intelligent tasks such as task-process optimization, manufacturing-resource optimization, and machine-design assurance in a CPS environment. Niggemann et al. [24] set out the challenges of a data-driven approach to control, monitor and diagnose cyber-physical systems, and proposed a cognitive architecture to address these challenges. They provided comparative schemas of the cognitive architecture and verified it by applying it to industrial case studies divided in the manufacturing site, energy analysis and big data analytics domains.

### 2.2. Big Data in Manufacturing

Research on big data is actively conducted in the manufacturing sector and in different sectors such as information, security, and business. Among these, big data-related studies that center on manufacturing industry have focused on the creation of data values through an analysis of big data and the resolution of issues related to the processing of massive data in the manufacturing sector. Chien et al. [25] applied big data analysis to determine the root cause of sub-batch processing systems in the semiconductor industry and proposed a big data-based framework that can analyze massive amounts of data generated in the semiconductor industry. Tekiner and Keane [26] proposed a big data architecture and framework composed of a system layer, a data collection layer, a processing layer, a modeling/statistical layer, a service/query/access layer, a visualization layer, and a data layer to manage massive amounts of data, and presented components of the sub-layers. Zhong et al. [27] designed and verified a framework that includes algorithms for the stages of data cleansing, compression and classification to classify and store radio frequency identification (RFID)-based logistic data and indicated the feasibility and practicality of storing and classifying big data. Zhang et al. [28] proposed an architecture for large data-driven analysis of the product lifecycle based on the product lifecycle management (PLM) perspective. They developed big data analysis focused on the manufacturing and maintenance process of the product lifecycle. Wan et al. [29] proposed a cloud-based system architecture for manufacturing big data systems in preventive maintenance, algorithms for real-time maintenance, and predictive management of data by analyzing the method of collecting data. Ji and Wang [30] proposed a big data analytics-based fault-prediction approach for shop-floor scheduling. They improved the availability of machining resources by mining potential fault/error patterns including machining errors, machining defects, or maintenance condition problems generated from the processing condition caused by an unsuitable schedule.

## 3. Cyber-Physical Production System (CPPS) Architecture Framework for Quality Prediction and Manufacturing Control

**Figure 1:**
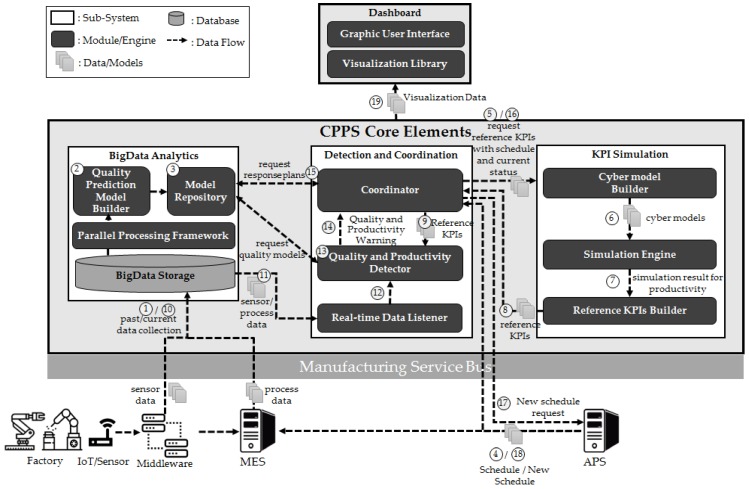
Cyber-physical production system (CPPS) architecture framework.

In this section, we present a CPPS architecture framework to predict the quality of metal casting and actively controlling manufacturing operations based on IoT, big data, AI, and simulation technologies; and we describe the operation mechanism of the entire systems by using the data flow between components. The CPPS analyzes data collected in real-time, predicts the quality and productivity of products, and supports the process of dynamically requesting rescheduling given the occurrence of irregularities by connecting information systems including the IoT, MES, and advanced planning and scheduling systems (APS). Figure 1 shows the entire CPPS architectural framework. The flow of data is noted in chronological order as indicated by the circle numbers. Specifically, ① to ⑨ correspond to the phase of building-up quality prediction models, response action models, and reference key performance indicator (KPI) models that should be performed prior to production, and ⑩ to ⑲ show the process of comparing the past or reference data based on real-time data after the start of production and actively changing the schedule when a problem occurs. Two circle numbers in an arrow denote that data is generated from the same data source, and data is reproduced in order after time.

The proposed CPPS is divided into three sub-systems, namely Big Data Analytics, a Detection and Coordination, and a KPI Simulation systems. Table 1 shows the explanations for CPPS core elements in detail.

Subsequently, the operating mechanism of the entire CPPS architecture framework is described by explaining the data flow between each module and sub-system as shown in Figure 1.

① Collection of past data: sensor and process data are collected through the IoT and MES and stored in the repository of the Big Data Analytics sub-system.
② Creation of quality prediction models: the collected data are analyzed by using AI techniques to create quality prediction models.
③ Storage of quality prediction/response models: the created quality prediction models and response plans are stored in the Model repository. The response plans and times are entered according to the operator’s experience.
④ Creation of production schedules: after production schedules are created in the APS, they are delivered to the Detection and Coordination sub-system and MES. Schedule data includes target outputs per machines or production lines. It could be specified to a timetable.
⑤ Request for reference KPIs: the Coordinator delivers the production schedules and current factory situation to the KPI Simulation sub-system and requests reference KPIs that should be used in the future as production-monitoring standards.
⑥ Creation of the cyber model: the Cyber model builder creates a cyber model for a KPI simulation based on the schedule and current factory situation.
⑦ Transmission of simulation results: after conducting a productivity simulation, the results are sent to the Reference KPI builder.
⑧ Transmission of reference KPIs: reference models of the production KPI values are created based on simulation results and sent to the Coordinator. The reference KPIs serves as the criteria for manufacturing execution monitoring and include target quality and production per unit time per process.
⑨ Loading of reference KPIs: reference models sent to the Coordinator are loaded into the Quality and productivity detector for the real-time monitoring of quality and productivity.
⑩ Real-time data collection: sensor and process data generated in the production process are collected in the Big Data Analytics sub-system.
⑪ Real-time data transmission: The collected sensor and process data are sent in real time to Detection and Coordination sub-system.
⑫ Real-time data monitoring: real-time data listener sends data collected in real time to the Quality and productivity detector.
⑬ Quality analysis and prediction: the Quality and productivity detector predicts the quality and productivity in real time by using the quality prediction model and the reference KPIs.
⑭ Transmission of quality/productivity irregularities: given the occurrence of irregularities related to quality/productivity, the corresponding information is sent to the Coordinator.
⑮ Request for analysis of response plans: the Coordinator requests the Model repository for analyses of response plans for irregularities and receives the corresponding information.
⑯ Request for future KPIs: in order to predict future KPIs caused by responses to irregularities, the current factory situation and production schedule is sent to the KPI Simulation sub-system. Additionally, KPIs are created through the same process as explained in points ⑥ and ⑧.
⑰ Request for new schedules: when the difference between the change in the initial reference KPIs and the future KPIs (due to irregularities) is significant, the Coordinator requests a new schedule to the APS.
⑱ Creation of new schedules: the APS creates new schedules by reflecting irregularities and sends them to the MES and Coordinator. Subsequently, it is repeated from ⑩.
⑲ Transmission of visualization information: when the above actions are performed, the progress of the entire systems is simultaneously sent to the dashboard in the form of visualization information that is visible to the user through the visualization library and graphical user interface.

## 4. Implementation and Case Study

This section describes the application and implementation of the CPPS architecture framework described in Section 3 to the metal-casting process of an actual piston engine factory. The purpose of the system is to predict the quality as soon as metal-casting process is completed and to actively change the production schedule after the machine is repaired if the number of defects exceeds the permissible number of defects. Section 4.1 explains the metal-casting process and quality issues of engine pistons. Section 4.2 describes the process of extracting features from the data collected from the IoT/MES by using Spark (which is a big data processing technology). Section 4.3 introduces the process of creating quality prediction models. Section 4.2 and Section 4.3 correspond to the Big Data Analytics sub-system in Figure 1. Section 4.4 explains the operation of quality problem detection, productivity simulation, and new schedule request based on a scenario by using a CPPS dashboard. It corresponds to the Detection and coordination sub-system and the KPI Simulation sub-system in Figure 1.

### 4.1. Metal-Casting Process and Quality Issues

**Figure 2:**
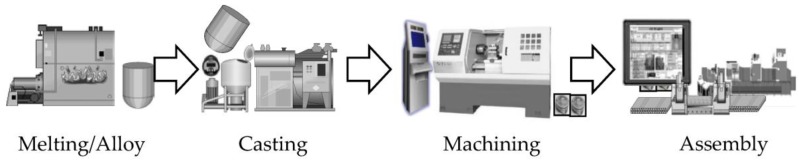
Overall process of the casting factory.

Given the characteristics of engine pistons, a permanent mold-casting method that employs metal molds is used in the target factory for mass production and precise management of the dimensions. First, the aluminium is heated and melted at high temperature inside the furnace, and additional elements are added to create an alloy. When the melting/alloy process is completed, the molten metal is divided into charge units, moved to the holding furnace of the specific casting line, and maintained in a stabilized state. Following this, the molten metal is injected into a casting machine and is transformed into casting after undergoing the solidification and cooling stages. The casting is transformed into a piston after the follow-up processes of heat treatment, surface treatment, machining, and assembly. Figure 2 summarizes the entire process of the target factory.

The main product defects in the target factory are generated in the metal-casting process. These defects are caused by various factors. Interview results revealed that excluding human errors, cold shuts and bubbles account for more than 90% of all metal-casting defects. However, it is not possible to detect metal-casting defects immediately after metal casting, and this is confirmed in subsequent processes. The reason it is difficult to confirm the defect is because it is a problem inside the product. The problem is only identified by cutting in the subsequent machining process. This implies that the products that should not be processed are machined. Considerable time and money is saved if the quality is predicted as soon as the metal-casting process is completed. The description of cold shuts and bubbles is given and their causes are summarized as follows.

- Cold shut: the defect occurs when the molten metal is poured into the mold and generates a boundary at the contact point where the molten metal joins. Generally, it occurs when the temperature of the molten metal is excessively low, when the pouring speed is excessively slow, or when the cross-section of the mold cavity is excessively thin.
- Bubble: the defect occurs when small bubbles are generated due to partial shrinkage cavities in the molten metal. Typically, bubbles are caused when the pouring speed of the molten metal is excessively fast, or when the solidification and cooling stages last an excessively long period after the molten metal is poured into the mold.

### 4.2. Data Collection

**Figure 3:**
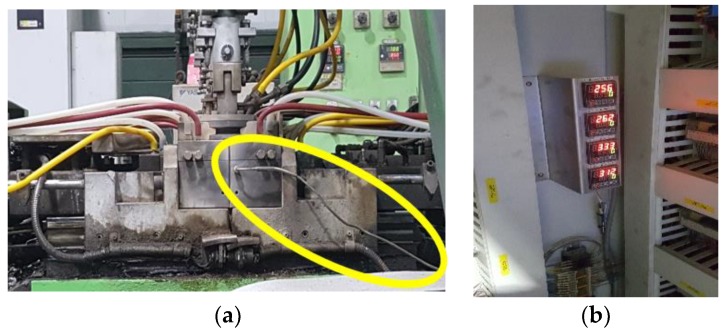
Installation of sensor and controller in the factory: (**a**) thermocouple sensor attached to the mold; (**b**) controller.

As summarized Section 4.1, the main causes of the casting defects have a lot to do with temperature. Therefore, we intended to collect and analyze temperature by attaching sensors to the mold in the casting machine to predict cold shuts and bubbles of products. Considering working temperature of casting process, we used k-type thermocouple (Chromel/Alumel) sensors that can measure a wide range of temperatures used in power plants or steel plants. Thermocouple sensors were installed on the mold and connected to the controllers to collect the temperature of casting process. Table 2 shows the specifications of the sensor. Figure 3a shows the sensor installed in the mold, and Figure 3b shows the controller connected to the sensors.

**Figure 4:**
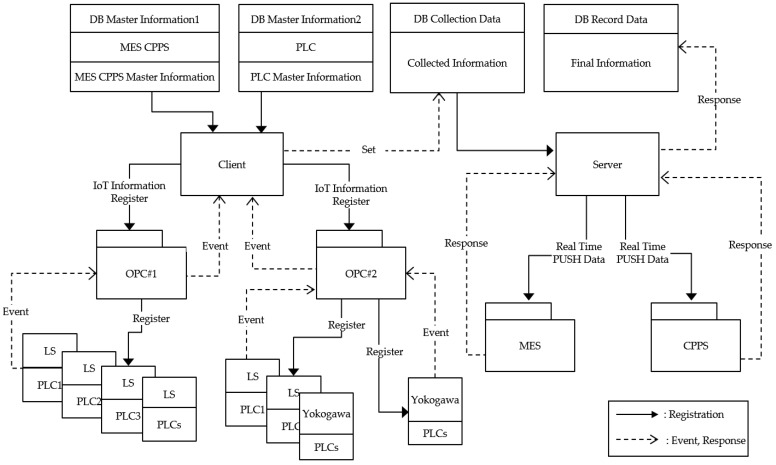
Interface of collecting data.

In addition to the temperature data, operational data such as the start and end times of the casting process are collected by each PLC (programmable logic controller) that controls the casting machines. Because existing PLCs of casting machines are different, we used open platform communication (OPC) which is well-known interoperability standard in industrial automation. If sensor data are generated, the data are aggregated through the OPC and are classified based on the master data of MES and CPPS. The aggregated data are sent in real time to the MES and CPPS, respectively. Figure 4 depicts the interface of sensor data. LS and Yokogawa are PLC manufacturing companies in Korea and Japan, respectively.

Data that are collected through IoT and MES to predict metal-casting defects are divided into holding furnace data, casting machine data, product data, and schedule data. Table 3 summarizes the collected data based on each category.

### 4.3. Pre-Processing Using Distributed Parallel Framework

**Figure 5:**
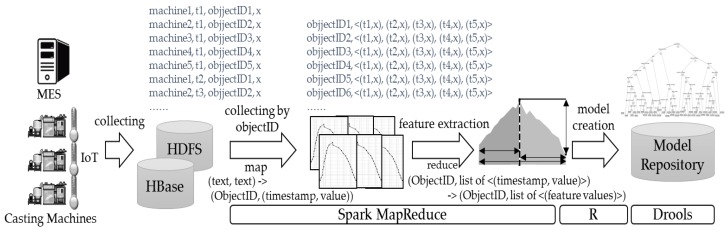
Example of MapReduce.

The IoT equipment installed in the casting machine and data obtained from MES are stored in HBase, which is a NoSQL database. Since the data collected through the IoT/sensors in the casting machine will be large-scale, storing it using the existing relational databases (RDB) is limited to considering the volume, velocity, and diversity of the big data [31]. For this reason, a NoSQL database supporting data distribution and parallel processing was chosen. All related data must be collected based on the product ID unit to create quality prediction models, and a feature extraction process is required to obtain time-series data of the mold temperature. In the study, Spark, which is widely used for high-speed data analysis, was used for the feature extraction. Figure 5 shows the process of feature extraction and prediction-model creation and storage. A Spark Map was used while collecting data based on the product ID units, and Reduce was used while extracting feature vectors from time-series data such as temperature.

Quality-prediction models are created through learning by using the appropriate classification algorithms on pre-processed data. In the study, R software was used to create quality prediction models, and different classification algorithms including a decision tree, random forest, artificial neural network, and support vector machine were used as the analysis techniques. The created quality prediction models are stored in a rule base (in the study, Drools 7 from JBoss was used), and finally utilized to identify defects by monitoring the data created in the IoT/MES in real time. Section 4.3 describes the process of creation of quality-prediction models in further detail.

### 4.4. Creation of Quality-Prediction Model

**Figure 6:**
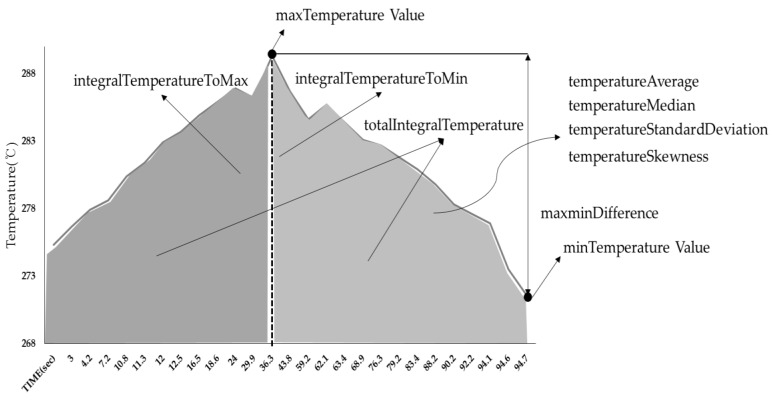
Extracting features from mold-temperature data.

The quality of metal casting is mainly affected by the variable corresponding to mold temperature. In order to generate a quality-prediction model, features must be extracted from metal-casting temperature data recorded by the time-series. Figure 6 describes the features extracted from the mold-temperature data.

The maximum/minimum mold-temperature values and the difference between them were extracted in conjunction with the cumulative temperature of the rising sections, cumulative temperature of the decreasing sections, and entire cumulative temperature values. In addition, the deviations and symmetry of the distribution were identified and included in the features by using the average, median, standard deviation, and distribution skewness of the entire section. Furthermore, differences exist in melting/alloying elements for each product, and thus they were included in the input variables used to create the prediction model. Table 4 shows all input variables used to create a quality-prediction model.

A dataset was created by collecting and pre-processing approximately three months of production data after constructing the CPPS. Table 5 shows the number of datasets of good-quality products, cold shuts, and bubbles based on the training and test sets used in the creation of the quality-prediction model.

In the study, we used R software to create the quality-prediction model. We used the Ctree library for the decision tree model, randomForest library for the random forest model, the nnet library for the artificial neural network model, and the e1071 for the support vector machine model. A training set was used to create the quality-prediction model, and a test set was used to evaluate the accuracy of the prediction. Table 6 shows the prediction results by using the decision tree, the random forest model, the artificial neural network model, and the support vector machine model. Table 7 shows a comparison of the prediction accuracy between all quality prediction models in Table 6. 

In summary, all prediction models except for the decision tree model showed a high level of prediction accuracy. Among them, the artificial neural network model showed the highest accuracy. However, comparing the average model-creation time, the artificial neural network model was found to require more time than the other models. Since detailed tuning was not done, it is likely that the accuracy and the generation time of each model will be able to be improved through proper model parameter tuning. As a result, it can be concluded that most classification models are suitable for predicting the quality of metal castings.

### 4.5. Problem Detection and Productivity Simulation

**Figure 7:**
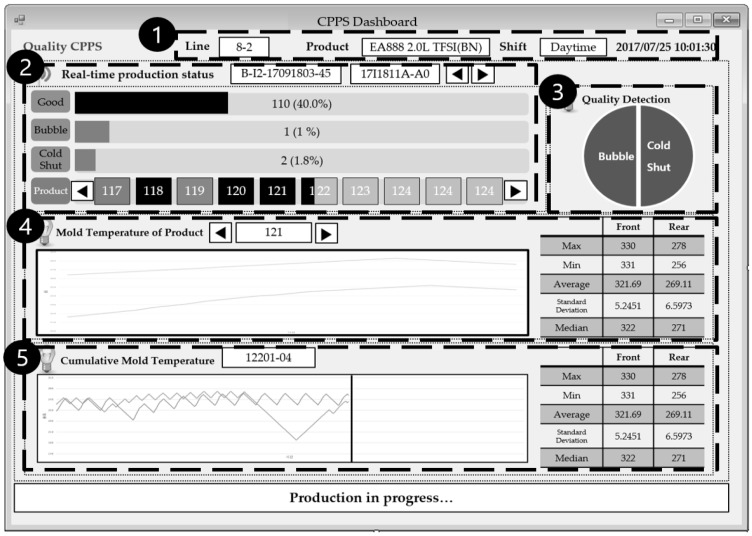
Main screen of the CPPS dashboard.

The section describes the process of quality problem detection, cyber model creation, productivity simulation, and new schedule request through a dashboard. Figure 7 shows the main screen of the dashboard, and Table 8 details each of its sections. The Detection and Coordination sub-system and the CPPS dashboard in the CPPS architecture presented in Section 3 were developed using C# language in NET Framework. In order to connect CPPS core components and MES/APS, RESTFull, which is a lightweight, maintainable, and scalable communication protocol in terms of heterogeneous applications, was used.

**Figure 8:**
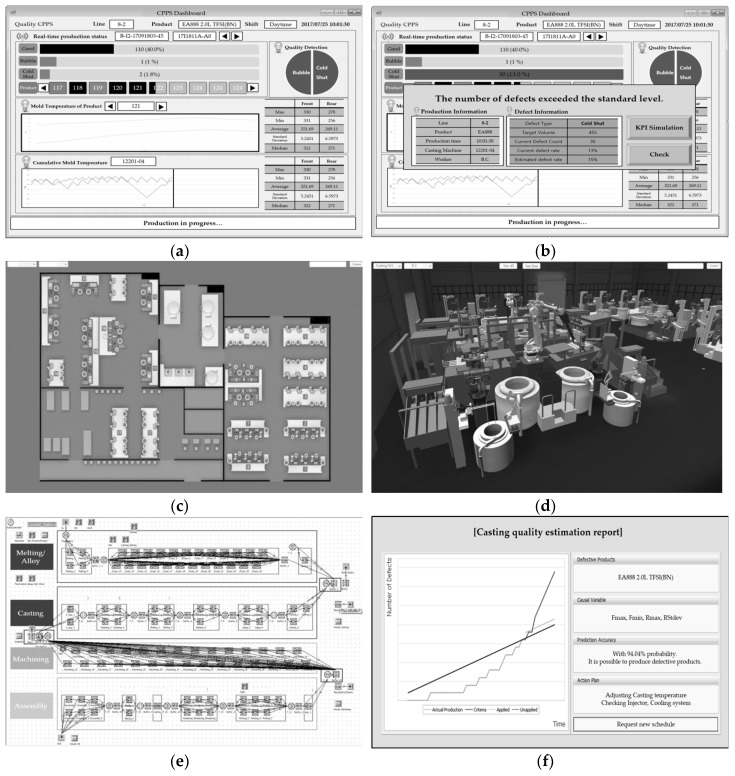
Screen shots of the CPPS dashboard: (**a**) screen shot for real-time monitoring; (**b**) screen shot for detecting defects; (**c**) screen shot for the factory layout; (**d**) screen shot of the 3D model for the casting line; (**e**) screen shot for the productivity simulation; (**f**) screen shot for requesting a new schedule.

As the production of a product progresses, the production status is updated in the dashboard in real time. It shows the existence of defects in each product, and the results of the defect-type prediction (Figure 8a). For this factory, if the cumulative number of quality defects exceeds the standard defect rate of 2% a lot, the dashboard emits a warning alarm and shows the user-summarized production and defect data (Figure 8b). After analyzing the detailed trends and variables that caused the defect, the user predicts changes in the KPIs related to production (such as the output, lead time and defect rate) by using a productivity simulation. (Figure 8c–e). Figure 8c shows the factory layout, and Figure 8d shows a selected manufacturing line modelled as 3D in the factory layout. Metal-casting machines are modeled by using Inventor, which is Autodesk’s 3D CAD program, and are synchronized to the physical factory in the Unity engine. Unity is a cross platform game engine developed by Unity Technology. It helps remote users in monitoring the manufacturing lines. Figure 8e shows a screen shot of a discrete simulation program for creating reference KPIs. For the productivity simulation, Plant simulation 11.0.3 of Siemens PLM Software (Plano, TX, USA) was used as simulation engine. Finally, the reference KPIs created prior to production are compared with the KPIs predicted through a simulation after several defects occurred, and the individual in charge requests a new schedule to the APS if the difference is significant (Figure 8f). For this factory, if (remaining hours) × (maximum output per hour) + (cumulative output) is much less than the daily target output, a new schedule is requested.

For one production line in the factory, the average monthly production is approximately 500,000, and the cycle time of subsequent process of the casting process was 37 s. Therefore, based on the number of monthly defect products which was approximately 10,000 (defect rate is about 2 percent), predicting 90% of the defect in the casting process can be concluded to save 92.5 h (37 s × 9000 = 333,000 s) of the time spent on subsequent processes. This corresponds to about 18.5% of the total monthly operating hours.

## 5. Conclusions and Future Work

Although factories in the past used specific automated production processes, future smart factories will evolve into autonomous adaptive next-generation plants that allow real-time production monitoring, real-time process optimization, customized flexible production, and real-time quality diagnosis and prediction by analyzing big data collected through the IoT. 

The study proposed an architecture framework to implement the cyber-physical production systems (CPPS) cooperating with other manufacturing information systems for quality prediction and operation control in metal-casting processes. It also described the process of applying and implementing the proposed CPPS architecture framework to change dynamic production schedules and predict quality defects in an actual piston-manufacturing plant. Finally, the proposed CPPS architecture framework was verified through the implementation of the CPPS dashboard based on a particular scenario. The CPPS monitors and predicts the occurrence of factory situations in real time based on the technologies such as the IoT, big data, and simulations. Furthermore, a CPPS acts as a coordinator to form optimal decisions related to the re-creation of production schedules through cooperation between the APS and MES. The solution presented in the study can predict the quality of the product as soon as the casting process is complete and reflect the process performance into the production schedule.

This study contributes to two key aspects related to CPS/CPPS implementation in manufacturing. First, this study shows technical examples of CPPS implementation in actual factories while research on CPS/CPPS is still in its infancy, and most published studies in popular journals are mainly focused on higher level architecture and reference models. In particular, this study presents more specific frameworks and use cases than previous studies, and introduced usable software and technologies. This can be referred to as a precedent implementation example for industry and academia considering CPPS implementation. Second, this paper presents the quantitative effect of implementing CPPS by estimating how much the subsequent process time can be reduced, even if estimated. It is expected that companies implementing smart factories up to automation and communication stage can reconsider their hesitation to build CPPS, and the result can have a positive impact on the industrial diffusion of CPPS.

Although the study addressed CPPS implementation with specific examples in manufacturing, a few issues persist with respect to CPPS implementation and diffusion. First, security issues in the exchange of various data should be considered. In the near future, a highly connected smart factory ecosystem across the global supply chain will be established by using CPPS. Security will become a bigger issue when various companies are connected. Second, the standardization of data formats and communication protocols among the various systems is required. There are several heterogeneous devices and applications in a factory. They increase the time and cost of system integration. The significance of this issue further increases if the factories are connected. Third, studies to reduce the resources needed for synchronization between a physical world and a virtual world are required. Most individuals expect a cyber model to correspond to a 3D model synchronized with the physical world. The creation of a 3D model and assignment of real-time data to predict the future consumes significant resources. With increases in the application range of CPPS, it is necessary to examine technical support for the synchronization of real-time data and real-time simulation of large-capacity 3D models.

## Figures and Tables

**Table 1:** CPPS core elements.

Sub-System	Component	Explanation
Big Data Analytics	Big data Storage	This stores big data generated from IoT/sensor and manufacturing execution systems (MES). Internet of Things (IoT)/sensor data may include raw sensory data, such as temperature, pressure, vibration, and process parameters, generated from machines and factories. Process data from MES may include input materials for each process, production status, location for work in process (WIP) and product, and quality inspection results. Considering that IoT/sensor data is large, NoSQL databases can be used to store real-time event data, and distributes file systems such as hadoop distributed file systems (HDFS) can be used for data analysis and old data storage.
	Quality prediction model builder	Data exploring and data preprocessing are performed through a parallel processing framework. Data learning, model generation, and model verification are performed using a machine-learning tool. Well-known parallel processing frameworks include Hadoop MapReduce and Spark. Mahout, Spark MLlib, R, and Python can be used as the machine-learning tools.
	Model repository	Building a machine-learning model is iterative because numerous machine-learning models are generated in order to meet some specific criteria. The model repository provides the ability to save and search machine-learning models. The machine-learning model can be saved as an XML-based format such as predictive model markup language (PMML). ModelDB, Microsoft Azure Machine Learning, and JBoss Drools can be used as the model repositories.
Detection and Coordination	Real-time data listener	This collects IoT/sensor and process data in real time. It may poll the event data stored in BigData Storage, and transmit it to the Quality and productivity detector.
	Quality and productivity detector	This detects quality and productivity problems of products in real time based on advanced planning and scheduling system (APS) schedules and quality prediction models. A warning is generated when the quality defect prediction amount exceeds the reference value or becomes smaller than the target production amount calculated by the APS.
	Coordinator	The module coordinates inputs and outputs between sub-systems and modules. The main roles are as follows. explore response plans and response times according to the problem; request reference KPI models such as production amounts and production times for the KPI simulation sub-system; request APS for new schedule considering response plan and current situation.
Key Performance Indicator (KPI) Simulation	Cyber model builder	This generates a cyber model (digital twin) based on real-time factory production status and the APS production schedule. The cyber models is synchronized to physical facilities, processes, systems, and factories. The cyber model has not yet been standardized. According to different purposes and level of details, the information that the cyber model can contain is very diverse. It can include geometries, structures, attributes, interfaces, rules, analysis models, and states. At the start of production or at the time of the occurrence of the problem, the cyber models update themselves from multiple sources, such as IoT and MES, to represent near real-time status, working condition, or position.
	Simulation engine	This carries out a productivity analysis using the cyber models created by the Cyber model builder. For example, 3D models created from computer aided design (CAD) software can be converted to simulation models by a simulation software. Then, productivity simulation is performed with parameters reflecting the status of the factory. The result of this module may include production amounts and times in the near future.
	Reference KPI builder	This creates reference KPI models to determine productivity problems based on simulation results. The result includes the target amount per hour of each machine which is calculated from the simulation result.

**Table 2:** Specification and image of the sensor.

Specification		Image
Thermocouple type	K	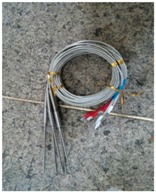
Line diameter Ø (mm)	3.2	
Service temperature (°C)	1000	
Maximum temperature (°C)	1200	

**Table 3:** Data classification of the casting process.

Categories	Data Name	Explanation
Holding Furnace	Holding Furnace ID	Identifier of the holding furnace in MES.
	Charge ID	Identifier of the charge in MES.
	Charge Element	Compositional elements of the charge.
Casting Machine	Casting Machine ID	Identifier of the casting machine.
	Front Mold Temperature	Temperature collected from the thermometer installed in the front of the mold.
	Rear Mold Temperature	Temperature collected from the thermometer installed in the rear of the mold.
Product (Piston)	Product Serial ID	Identifier of an individual product.
	Quality	Quality state of the products (good, cold shut, bubbling).
	Production Time	Production completion time of the product.
Schedule	Lot ID	Identifier of the lot in MES.
	Schedule	Schedule, such as production volume, time, and worker.

**Table 4:** Input variables for quality prediction.

Variables	Description	Source
Defect	defect type of product	MES
ProductID	ID of product	MES
FMax	max value in the front mold temperature section	IoT
FMin	min temperature value in the front mold temperature section	IoT
FStdev	standard deviation value in the front mold temperature section	IoT
FAverage	average temperature value of the front mold temperature section	IoT
FMedian	median temperature value in the front mold temperature section	IoT
FMax-Min	difference between max and min value in the front mold temperature section	IoT
FSkewness	skewness of the front mold temperature section	IoT
FintegralToMax	accumulated temperature value of the rising temperature zone in the front mold	IoT
FintegralToMin	accumulated temperature value of the falling temperature zone in the front mold	IoT
Ftotalintegral	accumulated temperature value of the total temperature zone in the front mold	IoT
RMax	max value in the rear mold temperature section	IoT
RMin	min temperature value in the rear mold temperature section	IoT
RStdev	standard deviation value in the rear mold temperature section	IoT
RAverage	average temperature value of the rear mold temperature section	IoT
RMedian	median temperature value in the rear mold temperature section	IoT
RMax-Min	difference between max and min value in the rear mold temperature section	IoT
RSkewness	skewness of the rear mold temperature section	IoT
RintegralToMax	accumulated temperature value of the rising temperature zone in the rear mold	IoT
RintegraloMin	accumulated temperature value of the falling temperature zone in the rear mold	IoT
Rtotalintegral	accumulated temperature value of the total temperature zone in the rear mold	IoT
ChargeElement	compositional elements of charge (IAL, AL2, UG, SIH, FE, CU, MN, MG, CR, NI, ZN, TI, CA, P, PB, SB, SN, SR, V, ZR, ALP)	MES

**Table 5:** Configuration of the dataset for constructing the quality-prediction model.

	Good	Cold Shut	Bubble
Training set	1047	978	970
Test set	482	386	416
Total	1529	1364	1386

**Table 6:** Prediction results of each quality-prediction model.

		**Predicted**					**Predicted**		
		**Good**	**Cold shut**	**Bubble**			**Good**	**Cold shut**	**Bubble**
Actual	Good	286	90	106	Actual	Good	421	38	23
	Cold shut	28	294	64		Cold shut	12	363	11
	Bubble	25	26	365		Bubble	7	4	405
(a) Decision tree model					(b) Random forest model				
		**Predicted**					**Predicted**		
		**Good**	**Cold shut**	**Bubble**			**Good**	**Cold shut**	**Bubble**
Actual	Good	442	21	19	Actual	Good	416	41	25
	Cold shut	10	367	9		Cold shut	18	359	9
	Bubble	6	4	406		Bubble	7	8	401
(c) Artificial neural network model					(d) Support vector machine model				

**Table 7:** Comparison of measurements between the quality-prediction models.

	Class	Precision	Recall	Overall Accuracy	Average Model Creating Time
Decision tree model	Good	0.8437	0.5934	0.7360	12 s
	Cold Shut	0.7171	0.7617		
	Bubble	0.6822	0.8774		
Random forest model	Good	0.9568	0.8734	0.9260	23 s
	Cold Shut	0.8963	0.9404		
	Bubble	0.9226	0.9736		
Artificial neural network model	Good	0.9643	0.8963	0.9384	1 m 27 s
	Cold Shut	0.9129	0.9508		
	Bubble	0.9355	0.9760		
Support vector machine model	Good	0.9433	0.8631	0.9159	21 s
	Cold Shut	0.8799	0.9301		
	Bubble	0.9218	0.9639		

**Table 8:** Description of each section of the main screen of the dashboard.

No.	Section Name	Explanation
1	Product Information	Information on target products.
2	Real-time Production Status	Product status expected by the prediction model.
3	Quality Detection	Expected defects.
4	Mold Temperature of a Product	Mold temperature of the latest product.
5	Cumulative Mold Temperature	Real-time mold temperature trend of casting machines.
